# Cancer imaging: Quo vadis?

**DOI:** 10.1186/1470-7330-14-1

**Published:** 2014-04-22

**Authors:** Rodney J Hicks, Kenneth A Miles

**Affiliations:** 1 Centre for Cancer Imaging, Peter MacCallum Cancer Centre, East Melbourne, VIC, Australia; 2 Institute of Nuclear Medicine, University College London, London, UK

## Editorial

At the launch of *Cancer Imaging*[1] on its new open access platform [2], our new editorial team welcomes readers and prospective authors.

When deciding how we are to move forward, like Janus, the Roman god of beginnings and transitions, it is also important that we look back on the successes and failures of the past. *Cancer Imagi*ng was, and remains, the journal of the International Cancer Imaging Society (ICIS) [3]. Initially founded as a forum for those in the radiology community with a sub-specialty focus on imaging cancer to share their experience, ICIS has also embraced specialists in nuclear medicine and radiation oncology. The centrepiece of its activities is an annual teaching course led by experts in various aspects of oncologic imaging. Fellows of the Society were asked to distil their experience into seminar and workshop presentations that were summarised in reviews of the best current evidence in their field and were published in a special supplement of *Cancer Imaging*. These articles were made available to participants in the course as a hard copy and subsequently online to members of the Society. Since Fellows of ICIS are only admitted by nomination and review of their track record of scientific contribution to the imaging of cancer, these reviews were highly respected by virtue of the reputation of the authors within their field of expertise. Accordingly, they have generally been well cited by other authors, leading to *Cancer Imaging* achieving both Pubmed listing and a respectable impact factor for a small and specialised imaging journal. Indeed its impact factor was higher than a number of better-known and much longer-established general imaging journals. Understandably, we are keen not to degrade this element of our publication. Towards this end, we have engaged an editorial board that has a high level of expertise in various specific domains of cancer imaging. We will be inviting each of them to commission review articles on a periodical basis to update advances in their field. Our focus will also be to enlist current and emerging opinion leaders to look beyond what is known and currently available to where there are exciting developments that should be the focus of new research.

As the reputation and visibility of *Cancer Imaging* increased, there was an increasing interest for both members of ICIS and other imaging specialists to publish original research articles in the journal. Extending the journal content to include original research would also help fulfil the society’s aim to stimulate research in the study of human tumour behaviour as well as promoting education in oncological imaging. While the prior publisher provided an opportunity for authors to publish their research as open access publications by payment of a fee, many opted not to pursue this option. This perhaps led to some excellent articles being less widely read and less often cited than they might otherwise have been. It is a natural hope of researchers that their peers will appreciate their hard work, observations and experience and many see citations as tangible evidence of this. By aiming to only accept original research articles that are of a high standard and guaranteeing that once published, all articles will be freely available, we expect that these articles will be read and cited more often.

As an entirely open access journal there are, of course, potential pitfalls. With a seemingly ever-expanding range of open access journals on ever more specialised subjects, one can wonder where medical publishing is heading and how this will impact the quality of the published literature. A case could be made that where a charge is levied on the author for publication of their article, there may be a financial incentive for the publisher to accept manuscripts regardless of quality whereas limiting publication to only the highest quality manuscripts is desirable when the costs of publishing are borne by the members of a society or a publisher relying on advertising revenue, which may be influenced by its impact factor. Indeed, in a recent celebrated example, a significant proportion of open access journals accepted a completely fabricated article. Therefore, convincing prospective authors to submit manuscripts with the kind of scientific rigour that will provide the journal with an ongoing reputation for quality and, in turn, enhance its impact factor, is a challenge for new entrants into the open access arena. Nevertheless, there are open access publishers that take the process of open access very seriously and it has been argued cogently that it is editorial process and not the manner in which publishing is financed that matters [4].

One of the great challenges for an editorial board, and particularly for an editor-in-chief, is to establish the benchmarks by which articles are judged. The noted statistician Martin Bland has argued that “bad statistics leads to bad research”. For the promotion of good research, the editors-in-chief will therefore seek to maintain statistical rigour for articles published in *Cancer Imaging*. For example, there will be an expectation for numerical observations, even those as apparently straightforward as diagnostic sensitivity and specificity, to be reported along with 95% confidence limits. Oncological imaging is a rapidly developing field and the editorial board anticipates a need to develop new benchmarks for judging articles in novel areas of research. One such example is the emergence of imaging biomarkers, a field in which even a cursory review of the imaging literature reveals lack of clarity both the use of nomenclature and the requirements for biomarker qualification. *Cancer Imaging* aims to be a leader amongst imaging journals in this regard by holding strictly to established definitions for prognostic, predictive and surrogate end-point (response) descriptors for biomarkers [5]. Furthermore, studies that use the same cohort to establish quantitative biomarker thresholds (cut-points) and assess their performance will be deemed as ‘exploratory’ unless appropriate cross-validation has been performed.

All members of our new editorial board [6] are highly published authors in their own right and know the frustration of having manuscripts rejected, particularly if reviewers were generally supportive in their comments or raised concerns that could be relatively easily addressed. Most of us have had reviews that we have felt to be unfair at best and incompetent at worst. We have also been involved as reviewers, volunteering our time to read manuscripts that while having redeeming features, need a great deal of polishing before they become fit for publication. It often takes much more time to review and critique a manuscript that is marginal for publication than it does for those that are clearly acceptable or not worthy of publication and the role of the responsible editor becomes much more fraught. In this regard, we will be highly reliant of the opinions and expertise of our section editors and will work with them to make the process of editing manuscripts as transparent as possible. Accordingly, when major revisions are required, we will seek to provide as much as advice and guidance as possible in meeting the requirements for publication. However, we must advise prospective authors that even their best efforts to address the concerns of reviewers and the section editor may not make the manuscript of sufficient quality and priority to lead to publication. While disappointing, we hope that this process of revision will have substantially improved the manuscript and make it more likely to find another suitable journal for publication. Further, we will hope to complete this in a timely manner, as we know how frustrating it is to have a manuscript rejected after months in review. We know that this wasn’t always achieved in the former life of *Cancer Imaging* and hope that prospective authors will give us the opportunity to demonstrate that the new editorial board and publisher will be able to deliver decisions expeditiously and allow rapid publication of articles that meet our requirements. In recommending *Cancer Imaging* as a journal that prospective authors should consider as a vehicle for communicating their original research or to share their experience in the application of imaging techniques within oncology, it is important that we, as Editors-in-Chief, can reassure them that our focus is on the highest principles of medical publishing.

Finally, it is the role of the Editor-in-Chief to determine the priorities regarding the type of research that should be published. With a plethora of imaging journals available to the would-be author or an imaging specialist trying to keep abreast of developments in their field, why would they choose Cancer Imaging? The first and most cogent reason it that it focuses on a disease that 1 in 2 males and 1 in 3 females will contract before the age of 85 and that represents the major cause of disability-adjusted life years lost in most developed countries. Additionally, imaging plays a pivotal role in the diagnosis of cancer but increasingly in the selection, planning and monitoring of cancer therapies. As healthcare costs are increasing, there are ever more stringent conditions for reimbursement being placed on investigations that are either perceived to be of high cost or that are being used more frequently than previously. If purely for the purposes of cost containment, this may be at the expense of patients’ rights to quality healthcare. Accordingly, we believe that we have a responsibility to cancer patients to promote the best possible investigations and to justify investment in imaging technologies to achieve optimal patient outcomes. However, we must also recognise that these need to be performed within the context of societal expectations regarding efficient use of fiscal resources. Therefore, we will strongly encourage manuscripts that address areas of clinical need in cancer management; that assess how imaging results impact management choices; or, that provide insights into cancer biology with prognostic and particularly, therapeutic implications. While there has been a long tradition of judging imaging tests by their sensitivity, specificity, negative and positive predictive values compared to a reference standard, such studies are often subject to selection bias in cancer populations. We will place a low priority on manuscripts that seek to compare one imaging test with another on these dimensions on the assumption that imaging tests are more often complementary than competing. We contend that more important questions relate to the sequencing of investigations and how best to use one or more tests to arrive at the optimal management plan in terms of both likelihood of efficacy and cost.

With these thoughts, we again welcome new and existing readers and authors to *Cancer Imaging*.

## References

[B1] Cancer Imaging http://www.cancerimagingjournal.com/

[B2] BioMed Central Open Access Charter http://www.biomedcentral.com/info/about/charter

[B3] International Cancer Imaging Society (ICIS) http://www.icimagingsociety.org.uk/

[B4] Letter to the Editor of Science, by Elizabeth Marincola http://blogs.plos.org/plos/2013/12/letter-to-the-editor-of-science-by-elizabeth-marincola/

[B5] Cancer Research Uk Biomarkers And Imaging Definitions 2014 http://www.cancerresearchuk.org/science/funding/apply/additional-information/biomarkers-imaging-definitions/

[B6] Cancer Imaging Editorial Board http://www.cancerimagingjournal.com/about/edboard

